# Aberrantly expressed GFRα-1/RET in patients with lacrimal adenoid cystic carcinoma is associated with high recurrence risk: a retrospective study of 51 LACC cases

**DOI:** 10.20892/j.issn.2095-3941.2020.0271

**Published:** 2021-02-15

**Authors:** Lin Liu, Liqiong Zhao, Jie Zhang, Guoxiang Song, Carol L. Shields, Ruihua Wei

**Affiliations:** 1 Tianjin International Joint Research and Development Centre of Ophthalmology and Vision Science, Eye Institute and School of Optometry, Tianjin Medical University Eye Hospital, Tianjin 300384, China; 2 Tianjin Orbit Institute, Ophthalmology Department, The Second Hospital of Tianjin Medical University, Tianjin 300211, China; 3 Ocular Oncology Service, Wills Eye Hospital, Thomas Jefferson University, Philadelphia 19107, PA, USA

**Keywords:** lacrimal adenoid cystic carcinoma (LACC), perineural invasion (PNI), GDNF, GFRα-1, RET, recurrence

## Abstract

**Objective:**

Because of the poor prognosis of lacrimal adenoid cystic carcinoma (LACC), we aimed to investigate the effects of perineural invasion (PNI) and consequent aberrations in GDNF/GFRα-1/RET protein expression on LACC recurrence.

**Methods:**

Clinicopathological data for 51 histologically confirmed patients with LACC enrolled between 2001 and 2017 were retrospectively analyzed. Hematoxylin and eosin staining was applied to assess PNI. Tissue-based immunohistochemistry (IHC) detection of GDNF, GFRα-1, and RET proteins was performed on LACC formalin-fixed, paraffin-embedded specimens. We generated semi-quantitative data of the IHC results and compared them with the clinicopathological data for the 51 patients.

**Results:**

Of the 51 patients, 19 (37.3%) were PNI positive. Recurrence was more common for LACC with than without PNI (73.7% *vs.* 37.5%, *P* = 0.01). GDNF, GFRα-1, and RET proteins were expressed in 62.7%, 62.7%, and 54.9% of the 51 patients with LACC, respectively. The expression of all 3 proteins was more common in patients with than without PNI. In agreement with previous findings, PNI-associated GFRα-1 and RET positivity, as detected by IHC, remained significantly associated with recurrence, whereas GDNF expression, as detected by IHC, was not correlated with LACC recurrence. Specifically, patients with concurrent GFRα-1 and RET expression may have a high risk of PNI (89.5% positivity rate) and recurrence (84.2% positivity rate).

**Conclusions:**

PNI may contribute to LACC recurrence. The concurrent expression of GFRα-1 and RET proteins, as detected by IHC, may potentially be associated with LACC PNI and recurrence.

## Introduction

Lacrimal adenoid cystic carcinoma (LACC) is the most common malignant subtype of lacrimal gland epithelial tumors^1,2^. Some LACC may derive from the accessory lacrimal glands of the conjunctiva^3^. Surgery and radiotherapy remain the standard therapeutic approaches for LACC^4,5^. Locoregional recurrence inevitably leads to disease progression and death^6,7^. Our previous study has suggested that, because of the anatomical location of the lacrimal glands, LACC can metastasize to the base of the skull, sinuses, and temporal fossa. The 5-year cumulative survival rate is 74.29%, and the mortality rate is 25.71%^8^. Therefore, molecular biomarkers and accurate detection are crucial for the prediction of LACC recurrence.

Perineural invasion (PNI), leading to metastasis to the base of the skull and to local recurrence, is an independent factor predictive of poor prognosis in head and neck adenoid cystic carcinoma^9^. However, the role of PNI in LACC recurrence has not been evaluated. Nerve growth factor signals, such as those of glial-derived neurotrophic factor (GDNF) and its receptors, are known to be involved in PNI of adenoid cystic carcinoma. GDNF, a member of the TGF-β family, was isolated, purified, and cloned in 1993^10^. Its receptor signal transduction complex comprises 2 parts: glycosylated phosphatidyl inositol anchored cell surface proteins, called GDNF family receptor α (GFRα), and Ret receptor of tyrosine kinase (RET). GFRα specifically binds GDNF family members, thus resulting in RET phosphorylation; phosphorylated RET in turn activates mitogen-activated protein kinase, thereby leading to the activation of a series of intracellular pathways. These signaling pathways provide a basis for the neurotrophic physiological function of the GDNF family^11–13^. However, the effects of GDNF/GFRα/RET expression on LACC remain unknown. Therefore, in the present study, we applied immunohistochemistry (IHC) methods to detect the expression of GDNF, GFRα-1, and RET in LACC specimens. After validation of the association between PNI and LACC recurrence, we conducted a correlative analysis for patients with LACC recurrence and positive GFRα-1 and RET expression. We present the first clinically significant demonstration of IHC-determined positive expression of GFRα-1 and RET in patients with LACC.

## Materials and methods

### Patients and pathology

**Figure 1:**
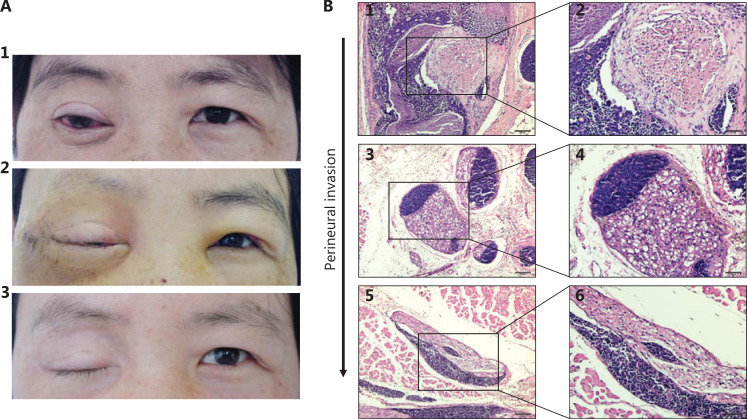
The representative photos of a patient and tissue section images of PNI with hematoxylin and eosin (H&E) staining. (A) A 39-year-old female with ACC in the right lacrimal gland. (A1) The patient had ptotic eyelid, conjunctiva congestion with pain and limited eye motility in all directions of gaze. (A2) The overview of this patient on the 13rd day after surgery: eyelid edema, conjunctiva edema and congestion, eye motility dysfunction. (A3) The overview of this patient after 4 months from the operation: total ptosis with skin scar. (B1 and B2) LACC cells grow around the nerve; (B3 and B4) LACC cells grow closer to the nerve; (B5 and B6) LACC cells invade into the nerve. B1, B3, B5 (H&E staining, 10 x); B2, B4, B6 (H&E staining, 20 x ).

The LACC specimens were obtained from recent surgical files. A total of 51 patients were enrolled between 2001 and 2017. All slides were examined by 2 pathologists. The pathological subtype (basaloid or non-basaloid) was determined according to the World Health Organization classification. All 51 patients with LACC provided informed consent to undergo the surgical procedure under a study protocol approved by Tianjin Medical University Eye Hospital review board [No. 2018KY(L)-15]. Formalin-fixed, paraffin-embedded (FFPE) specimens were stained with hematoxylin and eosin (H&E) to determine PNI. The results were reviewed by the 2 blinded pathologists. Tumor infiltration of the peripheral nerve sheath in a circumferential or partially circumferential pattern, or tumor growing through the nerve fibers was identified as PNI (**Figure 1**).

### Immunohistochemistry

FFPE tissue sections were stained with the streptavidin-biotin complex immune-peroxidase method. All FFPE sections of 5 µm thickness were prepared on charged glass slides. Tissue sections were deparaffinized and rehydrated, then treated with peroxidase buffer at room temperature. After antigen retrieval, the sections were incubated with anti-GDNF (BA0890, BOAO Biological Engineering Co. Wuhan, China, 1:100 dilution), anti-GFRα-1 (BS-0201R, Bioss Biological Technology, Beijing, China, 1:100 dilution), and anti-RET (BA1385, Boster Biological Technology, Wuhan, China, 1:100 dilution). All signals were detected with DAB staining.

### Cell proliferation assays

The ACC-2 cell line was purchased from the Wuhan Culture Collection (Wuhan City, Hubei Province, China) and cultured in RPMI 1640 with 10% FBS. All cells were maintained in a humidified 5% CO_2_ environment at 37 °C.

We plated 2,000 ACC-2 cells into each well of a 96-well plate for MTT assays. After the indicated time points, the MTT assays were performed by the addition of 20 µL of 5 µg/ml MTT to each well and subsequent incubation for 4 h at 37 °C.

### RNA extraction and PCR

Total RNA was isolated with TRIzol reagent (Invitrogen, Grand Island, NY, USA), then used for first strand cDNA synthesis and PCR (B639295, Sangon Biotech, China) according to the manufacturer’s protocol. The primers were as follows: GFRα-1, 5′ CCAGCCACATAACCACAAA 3′, 5′ AAGAGAACAGGAAACAGAT 3′; β -actin, 5′ ATCATGTTTGAGACCTTCAACA 3′, 5′CATCTCTTGCTCGAAGTCCA3′.

### Western blot

Total cellular protein was extracted with RIPA buffer. Then the prepared protein samples were loaded onto a gel. After electrophoresis, the protein was transferred to a PVDF membrane, which was then blocked with skim milk for 60 min. After washing and incubation with antibodies, the PVDF membrane was used for exposure and imaging.

### Statistical analysis

The chi-square test was applied to evaluate categorical variables. A two-sided *P* value < 0.05 was considered to indicate a statistically significant difference. All statistical analysis was performed in SPSS 25 statistical software (SPSS, IBM Corporation, Armonk, NY, USA).

## Results

### Patients with LACC and PNI may have high risk of recurrence

LACC he has been suggested to be malignant with high recurrence, and to have poor overall prognosis and controversial therapeutic approaches^14–17^. We hypothesized that LACC recurrence might be associated with PNI. To determine the potential correlation between recurrence and PNI, we enrolled 51 patients with LACC in our hospital. Representative photographs of a patient with eye-sparing surgery followed by adjuvant radiotherapy^17,18^ are presented in **Figure 1A**. Representative images of PNI with H&E staining are shown in **Figure 1B**. According to the H&E images, most tumor cells were located near nerves or had completely invaded into the nerves (**Figure 1B**).

A total of 19 patients (37.3%) were found to be PNI positive by 2 pathologists. According to the clinicopathological data of the patients with LACC, basaloid LACC tended to have more PNI than non-basaloid LACC (68.4% *vs.* 21.9%, *P* = 0.001). Interestingly, PNI was not associated with pain in patients with LACC (63.2% *vs.* 59.4%, *P* = 0.79). No significant differences in T stages between the 2 cohorts were identified (*P* = 0.81). However, recurrence was more common in LACC with than without PNI (73.7% *vs.* 37.5%, *P* = 0.01) (**Table 1**). The results together suggested that PNI might be correlated with recurrence in patients with LACC.

**Table 1:** Correlation of PNI with clinical and pathological characteristics in patients with LACC

Characteristics	Total	PNI (positive)	PNI (negative)	*P*
Cases, *n* (%)	51 (100.0)	19 (37.3)	32 (62.7)	–
Gender, *n* (%)				0.36
M	20 (39.2)	9 (47.4)	11 (34.4)	
F	31 (60.8)	10 (52.6)	21 (65.6)	
M: F	1: 1.6	1: 1.1	1: 1.9	
T stage, T1/T2/T3/T4, *n* (%)	51 (100)	2 (10.5)/4 (21.1)/6 (31.6)/7 (36.8)	4 (12.5)/10 (31.3)/10 (31.3)/8 (25.0)	0.81
Histopathological type, *n* (%)				0.001
Basaloid	20 (39.2)	13 (68.4)	7 (21.9)	
Non-basaloid	31 (60.8)	6 (31.6)	25 (78.1)	
Recurrence, *n* (%)				0.01
Yes	26 (51.0)	14 (73.7)	12 (37.5)	
No	25 (49.0)	5 (26.3)	20 (62.5)	
Pain, *n* (%)				0.79
Yes	31 (60.8)	12 (63.2)	19 (59.4)	
No	20 (39.2)	7 (36.8)	13 (40.6)	

PNI, perineural invasion; M, male; F, female. T stage, according to the American Joint committee on cancer (AJCC) classification 8th edition for lacrimal tumor staging.

### Highly expressed GFRα-1 and RET are associated with PNI and LACC recurrence

**Figure 2:**
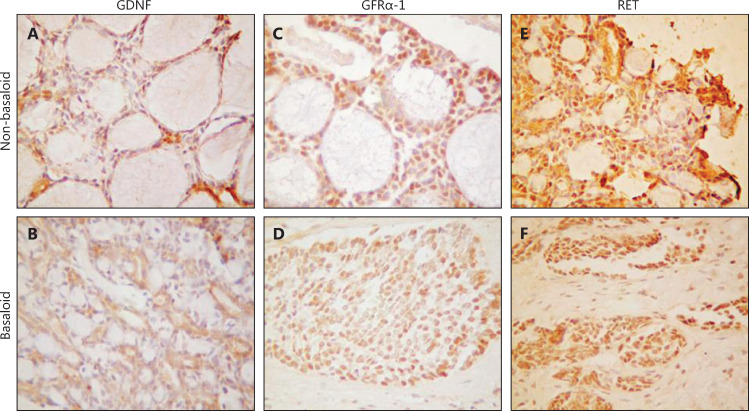
The representative images of GDNF, GFRα-1 and RET expression detected by immunohistochemistry (IHC). A: Positive expression of GDNF in the non-basaloid type of LACC; B: Positive expression of GDNF in the basaloid type of LACC; C: Positive expression of GFRα-1 in the non-basaloid type of LACC; D: Positive expression of GFRα-1 in the basaloid type of LACC; E: Positive expression of Ret in the non-basaloid type of LACC; F: Positive expression of Ret in the basaloid type of LACC. A, B, D, F (IHC staining, 20 x); C, E (IHC staining, 40 x).

GDNF has been well studied in other cancers and found to promote migration and invasion^19–22^. It is also involved in PNI in bile duct carcinoma^23^. GDNF signals including GFRα-1 and RET have been reported to function in PNI^19,24,25^. To investigate the role of GDNF and its receptors in LACC, we treated ACC-2 cells with GDNF. The results suggested upregulated proliferation of ACC-2 cells in a manner dependent on GDNF concentration and time, in treated cells compared with control cells (**Supplementary Figure S1A and S1B**). In agreement with findings from previous studies, the expression of GFRα-1 was dramatically upregulated by GDNF in ACC-2 cells (**Supplementary Figure S1C**)^11,12,26^. To investigate the roles of GDNF, GFRα-1, and RET in PNI, we determined the expression of these proteins in our FFPE LACC tissue samples through IHC. Representative images of GDNF, GFRα-1, and RET IHC detection are shown in **Figure 2**.

Subsequently, we generated IHC data from our LACC cohort. After examining the IHC results, we grouped samples into IHC positive and IHC negative, on the basis of pathological reports (**Table 2**). GDNF, GFRα-1, and Ret proteins were expressed in 62.7%, 62.7%, and 54.9%, respectively, of the 51 patients with LACC. Notably, PNI was more common in GFRα-1- or RET-positive patients (59.4% *vs.* 0, and 60.7% *vs.* 8.7%, respectively) than in negative patients. GFRα-1 or RET positivity was also associated with a high risk of recurrence (62.5% *vs.* 31.6%, and 64.3% *vs.* 34.8%, respectively). However, GDNF positivity was correlated with only PNI positivity and was not predictive of LACC recurrence. The levels of all 3 proteins did not significantly correlate with pain. Positive IHC detection of GFRα-1 or RET was associated with LACC PNI and recurrence.

**Table 2:** Correlation of GDNF, GFRα-1, and RET with clinical and pathological characteristics in patients with LACC

Characteristics	Total	GDNF		*P*	GFRα-1		*P*	RET		*P*
		+	–		+	–		+	–	
Cases, *n* (%)	51	32 (62.7)	19 (37.3)	–	32 (62.7)	19 (37.3)	–	28 (54.9)	23 (45.1)	–
PNI, *n* (%)										
Positive	19	19 (59.4)	0 (0.0)	<0.001	19 (59.4)	0 (0.0)	<0.001	17 (60.7)	2 (8.7)	<0.001
Negative	32	13 (40.6)	19 (100.0)		13 (40.6)	19 (100.0)		11 (39.3)	21 (91.3)	
Recurrence, *n* (%)										
Yes	26	14 (43.8)	12 (63.2)	0.18	20 (62.5)	6 (31.60)	0.03	18 (64.3)	8 (34.8)	0.04
No	25	18 (56.2)	7 (36.8)		12 (37.5)	13 (68.4)		10 (35.7)	15 (65.2)	
Pain, *n* (%)										
Yes	31	21 (65.6)	10 (52.6)	0.36	19 (59.4)	12 (63.2)	0.79	15 (53.6)	16 (69.6)	0.24
No	20	11 (34.4)	9 (47.4)		13 (40.6)	7 (36.8)		13 (46.4)	7 (30.4)	

GDNF, glial-derived neurotrophic factor; GFRα-1, GDNF family receptor α-1; RET, Ret receptor of tyrosine kinase; PNI, perineural invasion; IHC, immunohistochemistry; +, IHC-positive; –, IHC-negative.

### GFRα-1/RET positive patients have a high risk of recurrence

To evaluate the association between GFRα-1/RET and LACC recurrence, we examined the effects of concurrent GFRα-1 and RET positivity on PNI and recurrence. Interestingly, 17 of 19 (89.5%) patients with LACC and GFRα-1/RET positivity, as detected by IHC, had PNI. Similarly, 16 of 19 (84.2%) of patients with LACC and GFRα-1/RET positivity, as detected by IHC, had recurrence. Concurrent positivity for these 2 proteins was significantly more strongly associated with a high risk of PNI and recurrence than single protein positivity or concurrent negativity (**Table 3**).

**Table 3:** GFRα-1/RET positive patients have a high risk of recurrence

Gene	Total	IHC positive (+), IHC negative (–)						

GFRα-1		+	+		–		–	

RET		+	–	P*	+	P**	–	P***
Cases, *n* (%)	51	19 (37.3)	13 (25.5)	–	9 (17.6)	–	10 (19.6)	–
PNI, *n* (%)				<0.001		<0.001		<0.001
Positive	19	17 (89.5)	2 (15.4)		0 (0.0)		0 (0.0)	
Negative	32	2 (10.5)	11 (84.6)		9 (100.0)		10 (100.0)	
Recurrence, *n* (%)				0.004		0.003		0.032
Yes	26	16 (84.2)	4 (30.8)		2 (22.2)		4 (40.0)	
No	25	3 (15.8)	9 (69.2)		7 (77.8)		6 (60.0)	
Pain, *n* (%)				0.21		0.04		0.07
Yes	31	13 (68.4)	6 (46.2)		2 (22.2)		10 (100.0)	
No	20	6 (31.6)	7 (53.8)		7 (77.8)		0 (0.0)	

GDNF, glial-derived neurotrophic factor; GFRα-1, GDNF family receptor α-1; RET, Ret receptor of tyrosine kinase; PNI, perineural invasion; IHC, immunohistochemistry. P*, GFRα-1(+)/RET (+) *vs.* GFRα-1(+)/RET (–); P**, GFRα-1(+)/RET (+) *vs.* GFRα-1(–)/RET (+); P***, GFRα-1(+)/RET (+) *vs.* GFRα-1(–)/RET (–).

## Discussion

LACC grows slowly and has a variety of symptoms and signs, including pain; moreover, imaging studies may show peri- and intraneural infiltration^27^. Local recurrence and metastasis are very common, thus resulting in the poor prognosis of LACC^27,28^. In PNI, a prominent characteristic of LACC, cancer cells invade the surrounding nerves, thus providing an alternative route for metastatic spread. With para-neural infiltration, LACC cells break out of the nerve bundle membrane, grow rapidly along the nerve bundle, and may even invade surrounding normal tissues. Here, we demonstrated that PNI may lead to LACC recurrence, which is associated with poor prognosis^29^.

Previous studies of GDNF and its receptors in perineural invasive tumors have focused on pancreatic cancer, cholangiocarcinoma, and other hepatobiliary tumors. Veit et al.^30^ have found that GDNF has no effect on the proliferation of pancreatic cancer cell lines but can be used as an efficient inducer that enhances migration and invasion in pancreatic cancer cell lines. Ito et al.^31^ have suggested that pancreatic cancer cells express many RET proteins, which specially bind nerve tissue produced GDNF. GDNF, GFRα-1, and RET may promote PNI of LACC through complicated signaling processes, which require further investigation. In this study, we aimed to develop an effective biomarker approach to estimate the risk of LACC recurrence through tissue based IHC detection. This study reports the first detection of all 3 proteins in LACC tumors by IHC and the determination of their association with LACC clinical data. Understanding of the underlying molecular mechanisms of these proteins in LACC is limited, and therefore further investigation is ongoing.

The present study used an IHC method to determine the expression of GDNF, GFRα-1, and RET in 51 LACC FFPE tissues and found positivity rates of 62.7% (32/51), 62.7% (32/51), and 54.9% (28/51), respectively. Patients with GFRα-1 or RET expression, as determined by IHC, particularly GFRα-1(+)/Ret (+) patients, tended to have a high risk of recurrence, thus suggesting that GFRα-1/Ret IHC detection may be a potentially effective approach to predict LACC recurrence in clinical settings. Our results suggest that the expression of GDNF, GFRα-1, and Ret is not correlated with pain in LACC. However, our study is limited by the small patient cohort and the signal detection method.
