# Supplementary material for: Aberrantly expressed GFRα-1/RET in patients with lacrimal adenoid cystic carcinoma is associated with high recurrence risk: a retrospective study of 51 LACC cases
Source: Cancer Biol Med. 2021 Feb 15;18(1):199–205. doi: 10.20892/j.issn.2095-3941.2020.0271 (PMC7877180; doi:10.20892/j.issn.2095-3941.2020.0271)
Supplement: Supplementary file 1 [file cbm-18-199-s001.pdf]

Supplementary material

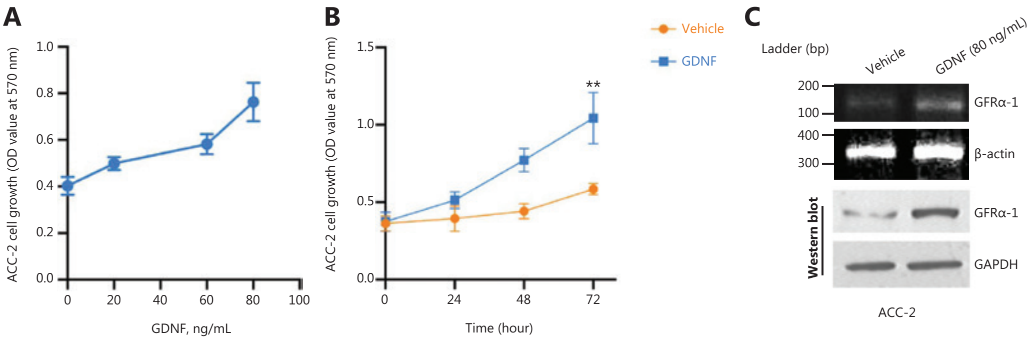

Figure S1 A. ACC-2 cells growth curve with different concentrations of GDNF (ng/mL) treatment for 48 h. B. ACC-2 cells growth curve with 80 ng/mL GDNF treatment for 72 h. C. PCR and western blot detection of GFRα-1 expression in ACC-2 cells cultured with GDNF (80 ng/mL).
